# Straw Mulch Induced Indoleamines Alleviate Reproductive Depression in Cold Sensitive Hazelnut Cultivars

**DOI:** 10.3390/plants12132577

**Published:** 2023-07-07

**Authors:** Murali-Mohan Ayyanath, Mukund R. Shukla, Yasmine S. Hezema, Praveen K. Saxena

**Affiliations:** 1 Department of Plant Agriculture, Gosling Research Institute for Plant Preservation, University of Guelph, Guelph, ON N1G 2W1, Canada; ayyanath@uoguelph.ca (M.-M.A.); mshukla@uoguelph.ca (M.R.S.); yhezema@uoguelph.ca (Y.S.H.); 2 Department of Horticulture, Damanhour University, Damanhour, El-Beheira 22713, Egypt

**Keywords:** hazelnut, phenology, cold stress, indoleamine toolkit, straw mulch

## Abstract

Climate change is forcing physiological changes, especially in temperate trees, in which the reproduction phase has been affected harshly, eventually resulting in poor performance. Erratic fluctuations during the flowering periods, predominantly in cold-sensitive, yet industry-desired (sourced), hazelnut cultivars have been causing at least a 10-fold decline in the nut yield. Indoleamines have been noted to provide protection during such abiotic stress conditions. In this study, we investigated the potential involvement of the indoleamine pathway in countering reproductive depression in cold-sensitive hazelnuts by blanketing the ground with wheat straw mulch. The female flower ratio; titers of tryptophan, serotonin, and melatonin; and indoleamine pathway gene regulation were the endpoints for assessing the effects of straw mulch. In the preceding year, we noted that the occurrence of phenological events through the modulation of indoleamines was necessitated via percolation of snowmelt into the rootzone. Otherwise, reproductive depression was noted, especially in harsh conditions, such as ‘no snow’ or when the rootzone was covered with a plastic sheet to disallow water percolation. When cold-sensitive hazelnut cultivars that were subjected to such deleterious treatments in the preceding years’ experiments were treated with straw mulch, the female flower ratio was unaffected and remained on par with that of the cold-hardy locally adapted cultivars. Tryptophan accumulation improved in the (cold-sensitive) sourced cultivars treated with straw mulch and was available as serotonin to counter the cold stress. Lower titers of melatonin explained the slight improvement in female ratio in the sourced cultivars blanketed with straw mulch. *ASMT* gene regulation via straw mulch treatment emphasized its role in abiotic stress mitigation. A negative trend was noted when improved flowering was compared to the decreased expression of the *ASMT* gene. Horticultural changes, such as mulch, should provide mitigating solutions to relieve reproductive depression in cold-sensitive hazelnuts, alongside implications in other horticultural crops. The indoleamine toolkit (cellular markers) developed in this study provides insights into the mechanisms of cold sensitivity (abiotic stress) and plausible solutions, such as exogenous application of indoleamines, to propagate climate resilient plant materials with an enhanced capacity to mitigate abiotic stress conditions.

## 1. Introduction

Climate change is driving unpredictable changes in temperature and water availability, affecting the adaptability of plant species in adverse environmental conditions, such as extreme cold and heat. Phytohormones, especially indoleamines, for example, melatonin (MEL) and serotonin (SER), play vital roles in alleviating abiotic stress while improving growth parameters, such as flowering [1]. Indoleamines have recently emerged as essential stress-mitigating compounds in plants. The presence of MEL in living medicinal plant tissues has elucidated the MEL biosynthetic pathway in plants and their roles in plant morphogenesis, reproduction, and stress survival [2]. MEL and SER also act as potent antioxidants, both directly detoxifying reactive species, and through the mediation of other antioxidant systems. MEL improves growth and survival under extreme (cryopreservation at −196 °C) [3] to moderate stresses (4 °C) [4] in diverse species. MEL and SER labeled using Quantum Dot nanoparticles for real-time tracking in living tissues showed that thermal stress disrupted normal localization of MEL and SER and dispersed them across cells, suggesting that plants have a mechanism to utilize these molecules throughout their tissues as antioxidants for protection against stress [4]. MEL upregulated the expression of several cold-responsive and cold-stress-related transcription factors (for example, *CBF/DREB*s, *COR15a*, *CAMTA1*, and *ZAT10/12*), in response to cold stress in *Arabidopsis thaliana* [5]. MEL-mediated improvements in stress survival have additionally been indicated in a diversity of species in which they function to upregulate diverse plant growth regulation biosynthetic pathways and signaling and defense networks to mitigate the effects of cold stress [5,6,7].

Tryptophan (TRP), an essential aromatic amino acid, is one of the building blocks for protein synthesis and the source of many important compounds, particularly MEL and nicotinamide adenine dinucleotide in animals [8] and indole-3-acetic acid and phytoalexins in plants [9]. In plants, TRP has been shown to affect various long-term physiological processes, such as organ growth, responses to stress and pathogen elicitors, and tolerance of heavy metals [10]. Nevertheless, little has been reported about the mechanism by which TRP affects growth and development at the cellular and molecular levels [11]. Recent research has demonstrated that indoleamines may modulate plant responses via a symphony of actions of molecules across the entire indoleamine pathway rather than a specific molecule [12]. These indoleamines are commonly synthesized from TRP via its conversion into SER and eventually leading to MEL [2]. TRP serves as a common precursor for the biosynthesis of SER and MEL, as well as the classical phytohormone auxin. In plants, of the several proposed pathways, it appears that the indoleamines are synthesized from TRP as it is first converted to tryptamine (TRM) by tryptophan decarboxylase (*TDC*), which is an aromatic amino acid decarboxylase in plants [13]. After the conversion of TRP to TRM, tryptamine-5-hydroxylase (*T5H*) then converts TRM to serotonin (SER). SER is then acetylated by serotonin-N-acetyltransferase (*SNAT*) to form N-acetylserotonin (NAS), which is finally converted to MEL by acetylserotonin-O-methyltransferase (*ASMT*) [14,15]. Plant defense and acclimatization to abiotic stress are related to the spatial and temporal distribution of SER and MEL [4]. Upregulation of MEL biosynthesis is expected to occur under stress conditions, thus indicating the antistress properties of MEL. 

Temperate trees, such as hazelnuts, present a good example of the influence of fluctuating cold temperature on adaptation, survival, and reproduction. Hazelnut is a monoecious species with female and male reproductive structures on the same branch that do not mature synchronously. The meager information available on the reproductive physiology of this species makes it difficult to understand and determine its performance globally. The male reproductive organs, the catkins, are short lived and sensitive to frost and fluctuations between warm and cold weather, which are quite common during the early spring in colder regions such as Ontario, Canada. This climate is exceptionally damaging for commonly cultivated, cold-sensitive, European cultivars of hazelnut (*Corylus avellana* L.) and may lead to rapid pollen dehiscence, in addition to a decline in the female flower ratio. These cultivars, referred to as the sourced cultivars when cultivated in a different climatic zone, are desirable and economically important for industry and growers engaged in the production of hazelnut-based products [16]. However, the cultivation of sourced cultivars is often severely restricted due to their inability to adapt to foreign environments, which affect the survival of flowers and catkins due to erratically fluctuating weather conditions [16,17]. The poor performance of sourced cultivars might result from the longer required time for acclimation and entering the reproduction phase, in addition to reallocating energy toward defense against cold because of cold susceptibility during the initial establishment phase [16].

**Figure 1:**
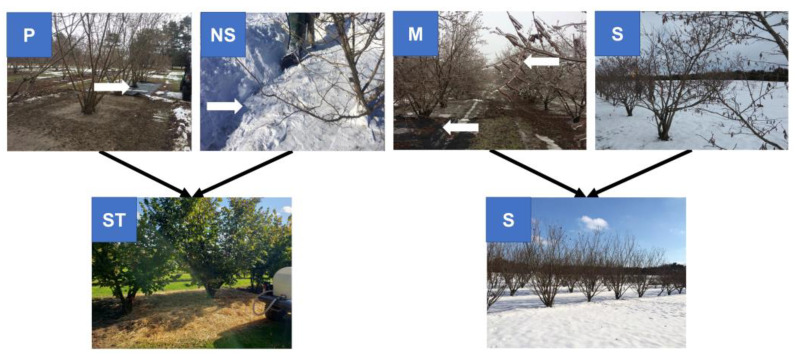
Schematic diagram depicting 2019 experimental design [17] in top panel, with P (plastic sheet that disallows snowmelt/water percolation), NS (no-snow; snow removed periodically until the following spring), M (porous plastic mulch sheet that allows water percolation), and S (naturally occurring snowfall); and 2020 experimental design in the bottom panel, with ST (wheat straw was applied before the trees entered dormancy, and this treatment included the trees from the P and NS treatments of the 2019 design) and S (naturally occurring snowfall, and this treatment included the trees from the M and S treatments of the 2019 design). Arrows in white are pointing at the treatments and a second arrow in M points at frozen catkins.

Our previous (Figure 1) study illustrated that snowmelt percolation was more significant to cold stress mitigation than the thermal effect [17]. Additionally, water percolation alleviated cold stress in hazelnut cultivars by modulating SER and MEL content in the reproductive tissues and consequently affected flowering and yield. In that study [17], poor female flower percentage was noted when the sourced cultivars were exposed to continuous removal of snow (NS) and plastic sheet (P) treatments, presumed to be the harshest treatments due to the absence of snowmelt or disallowing of snowmelt percolation into the ground during the flowering period. Therefore, we hypothesized that these treatments influenced flowering, which probably could be explained by the regulation of genes involved in the indoleamine biosynthesis pathway. To advance that study, we further hypothesized that blanketing the harsh treatments (NS and P) with straw mulch while the trees entered endodormancy would improve the flowering percentage in the subsequent year compared to natural snow conditions. Wheat straw is a cheaply available ground cover material that provides thermal protection and allows water flow [18,19,20]. We hypothesized that straw mulch, with its thermal and water percolation regulation potential, might alleviate cold stress and modulate flowering in the sourced cultivars via stress-busting indoleamine titers and the associated genetic expression of the pathway. Specifically, biochemical endpoints, such as TRP, SER, and MEL titers, in addition to the expression of selected indoleamine biosynthesis pathway-related genes in male and female reproductive organs obtained during dormancy and during flowering, were assessed. Here, we hypothesized that the accumulation and metabolism of TRP, SER, and MEL via indoleamine pathway-related genes could modulate the development of vegetative and flowering buds. The results of the present study demonstrated that the reproductive depression and decline in the yield of sourced hazelnut trees due the absence of thermal insulation provided by snow cover and alleviation of stress are both mediated by interplay of the indoleamine pathway. These findings have practical applications as plausible horticultural solutions to support established trees in the ground against fluctuating cold and warm spells, as well as the assessment of the cold tolerance potential of novel germplasm before transplantation. The simple yet highly effective cold stress protection strategies mediated by ubiquitous indoleamines, as described in this study, could be extrapolated to other commercially important horticulture germplasm, including those that need to be relocated to new agroclimatic zones.

## 2. Results

Hazelnut trees transplanted in 2008/2009, belonging to 15 cold-sensitive cultivars (premium sourced cultivars) in parallel with locally adapted cultivars, were grown over a year with the following treatments: (a) wheat straw mulch (extra blanket under the snow); and (b) natural snow. Developmental, biochemical, and molecular analyses were performed on reproductive organs (dormant and flowering) and vegetative buds. 

### 2.1. Reproductive Phase Development in Hazelnut

**Figure 2:**
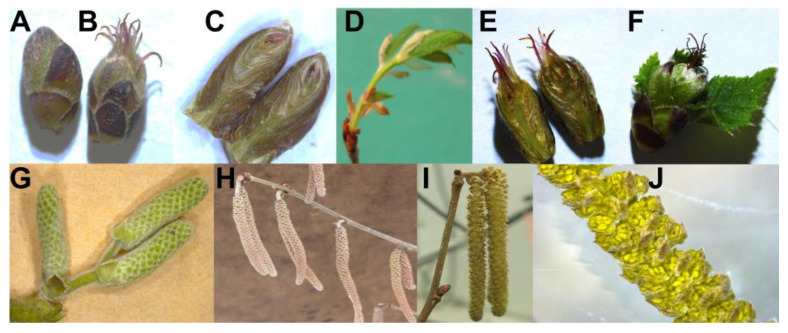
Reproductive phase development in hazelnut: a dormant bud either (**A**) turns into a female flower (**B**) in winter as each bud has potential to develop into a female flower (**C**) before the vegetative bud breaks or (**D**) directly turns to the vegetative phase without developing into a female. The female flower (**E**) has multiple pistils that are receptive to pollen for effective fertilization and nut set (**F**). The young male flower ((**G**), catkins) develops during the previous year’s growth cycle, and mature catkins (**H**) remain dormant until mid-winter. The healthy catkins elongate (**I**), while anthers (**J**) dehisce pollen.

Typically, female flowers emerge during end of the winter in the continental climate of southern Ontario, and later vegetative bud breaking starts [16]. Each dormant bud has potential to become a female flower, and the female flower-to-vegetative bud ratio depends upon several factors, such as genotype, location, and weather conditions. Each bud possesses the needed gear to become a female flower (Figure 2). Hazelnut cultivars can be either protandrous or protogynous depending on the cultivar. The catkins elongate and mature over a period and start to dehisce for a few days in congenial conditions.

### 2.2. Phenology

**Figure 3:**
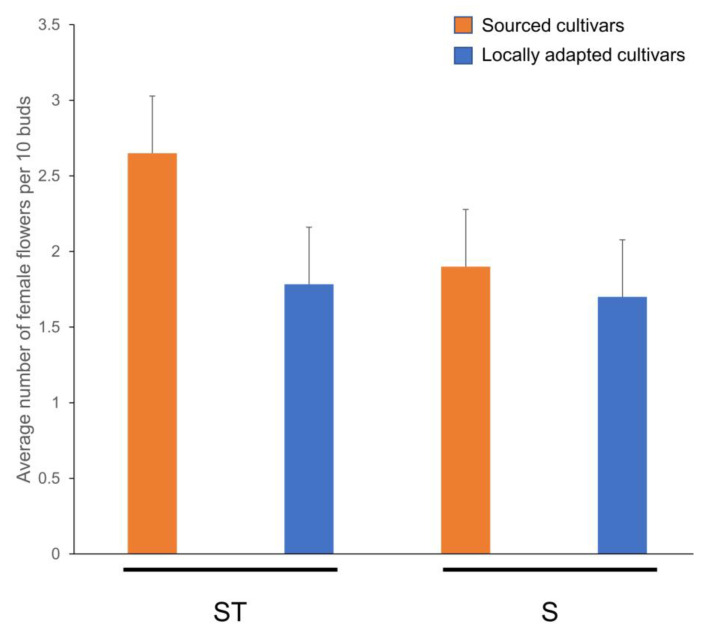
Alleviation of reproductive depression in cold-sensitive hazelnut cultivars using straw mulch. Labels on the x-axis, ST and S represent wheat straw mulch and naturally occurring snow treatments, respectively. Error bars represent SEMs.

There were no statistical differences between the interactions of cultivars and the treatments (*p* _(1,20)_ = 0.39). However, further to our anticipation of no significant differences in the flowering responses among the interactions, interesting trends were noted in which a better female flower percentage was noted in the sourced cultivars blanketed with straw mulch (Figure 3). The female flower percentage of locally adapted cultivars remained unaffected by the treatment. 

### 2.3. Separation of Metabolites and Metabolite Detection (UPLC-MS)—Snowmelt Percolation Study

The effects of M, NS, P, and S treatments on the changes in indoleamine titers of sourced and locally adapted hazelnut cultivars were described elsewhere [17]. To summarize, accumulation of SER and MEL titers in NS and P treatments appeared to decrease the flowering percentage in sourced hazelnut cultivars. Later, these treatments were merged to be replaced with straw mulch before it snowed in late fall. 

### 2.4. Separation of Metabolites and Metabolite Detection (UPLC-MS)—Straw Mulch Study

**Figure 4:**
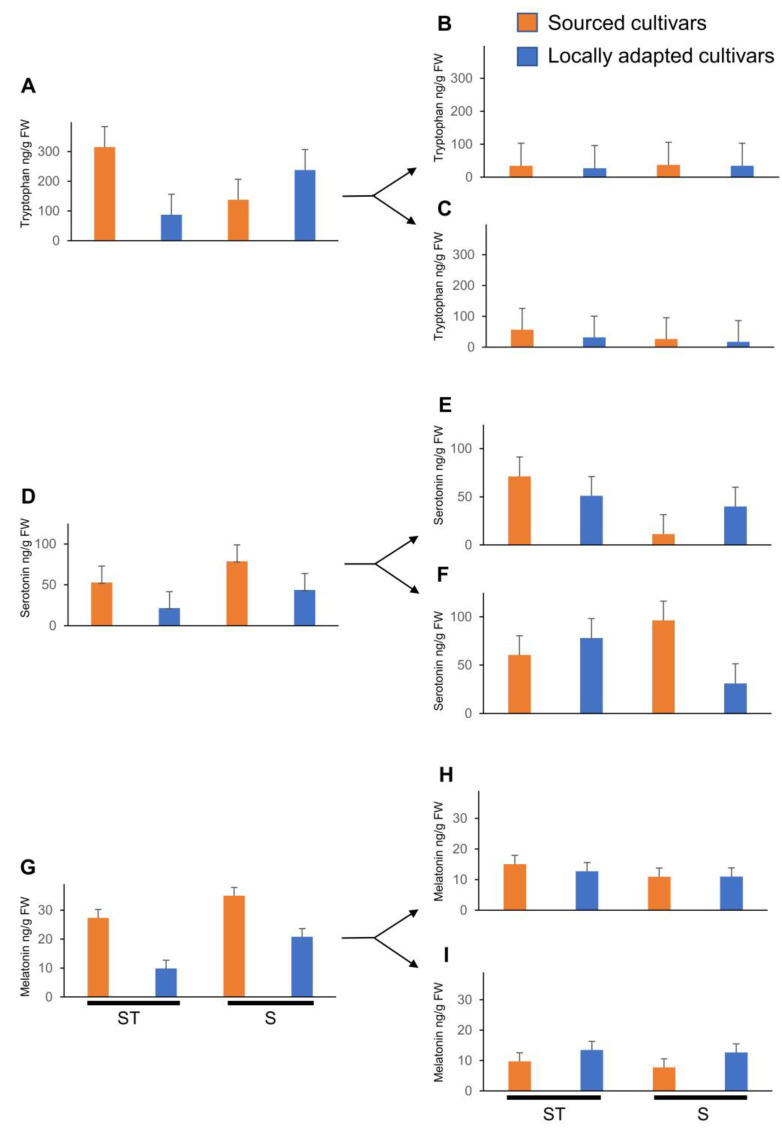
Effect of cold stress (abiotic) on indoleamine metabolite concentrations, viz. tryptophan, serotonin, and melatonin, in dormant ((**A**,**D**,**G**), respectively; initiated arrows), vegetative ((**B**,**E**,**H**), respectively; diverged up arrow), and flowering buds ((**C**,**F**,**I**), respectively; diverged down arrow) of sourced and locally adapted hazelnuts when exposed wheat straw mulch and natural snow conditions. Labels on the x-axis, ST and S represent wheat straw mulch and naturally occurring snow treatments, respectively. Error bars represent SEMs (ng/g FW) for that compound.

When TRP concentrations were analyzed for the effects of treatments (straw or snow), cultivars (locally adapted or sourced), and kind of bud (dormant bud or catkin, dehiscing catkin, female flower, or vegetative bud), statistical significance was noted in overall interactions (*p* _(4,100)_ < 0.0001), in kind of bud and cultivar interaction (*p* _(4,100)_ = 0.0023), and in kind of bud and treatment interaction (*p* _(1,100)_ < 0.0001). Dormant buds of sourced cultivars accumulated at least 3.5 times higher TRP titers when blanketed with straw mulch compared to locally adapted cultivars (Figure 4A). There was no difference in the TRP titers of dormant buds between the cultivars when they were exposed to natural snow conditions. A similar trend was noted between natural snow- and straw mulch-exposed sourced cultivar flowers, whereas the trend was reversed in the flowers of locally adapted cultivars. It is noteworthy that the TRP titers remained unchanged among the cultivars or treatments in the vegetative buds.

**Figure 5:**
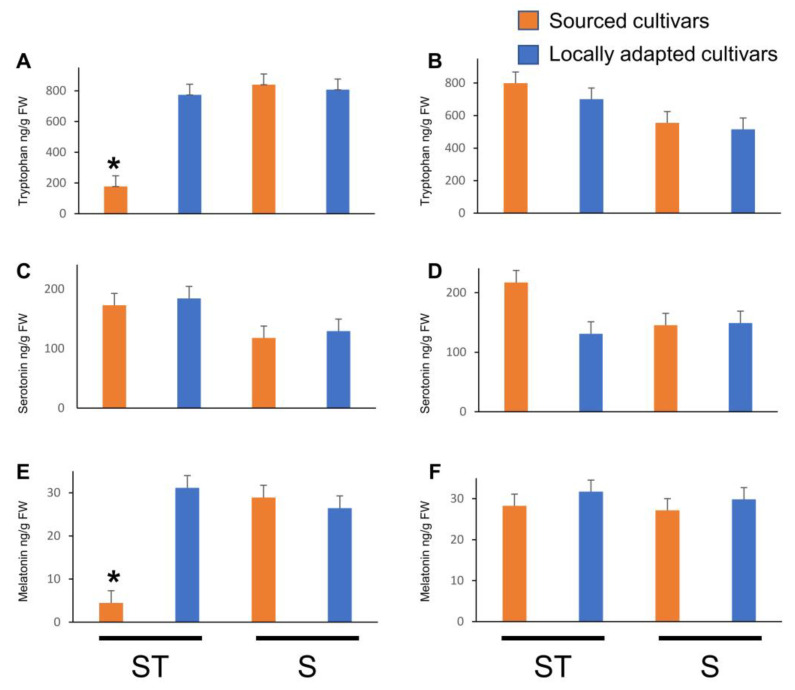
Effect of cold stress (abiotic) on indoleamine metabolite concentrations, viz tryptophan, serotonin, and melatonin, in dormant ((**A**,**C**,**E**), respectively) and dehiscing ((**B**,**D**,**F**), respectively) catkins of sourced and locally adapted hazelnuts when exposed wheat straw mulch and natural snow conditions. * denotes statistical significance in responses compared between the treatment and control groups (*p* < 0.05), and error bars represent SEMs (ng/g FW) for that compound. Labels on the x-axis, ST and S represent wheat straw mulch and naturally occurring snow treatments, respectively.

Titers of TRP in dormant catkins were at least 2-fold lower in natural snow conditions, but an opposite trend was noted in the dehiscing catkins, in which straw mulch resulted in higher TRP titers (Figure 5A). Dormant catkins of locally adapted cultivars possessed ca. 1.5-fold higher TRP titers than the sourced cultivars. However, there were no such differences between the cultivars during the dehiscing stage. When exposed to straw mulch, the dormant catkins of sourced cultivars possessed at least 4- to 5-fold lower TRP titers compared to other treatment and cultivar combinations. It is interesting to note that the opposite trend occurred in the dehiscing catkins of sourced cultivars that were exposed to straw mulch. 

When SER concentrations were analyzed for the effects of treatments (straw or snow), cultivars (locally adapted or sourced), and kind of bud (dormant bud or catkin, dehiscing catkin, female flower, or vegetative bud), statistical significance was noted in overall interactions (*p* _(4,100)_ = 0.0393), kind of bud (*p* _(4,100)_ < 0.0001), and treatment (*p* _(1,100)_ = 0.0291). However, the interaction between kind of bud and treatment was near significant (*p* _(4,100)_ = 0.0653). Dormant buds of sourced cultivars exposed to natural snow conditions accumulated at least 3.5-times higher TRP titers compared to locally adapted cultivars blanketed with straw mulch (Figure 4A). In the flowers of sourced cultivars exposed to natural snow conditions there were ca. 3-fold higher titers of SER compared to locally adapted cultivars. When the sourced cultivars were blanketed with straw mulch, ca. 6-fold higher SER titers in the vegetative buds were noted compared to those exposed to natural snow conditions. Titers of SER in dormant catkins were at least 1.5-fold higher in locally adapted cultivars exposed to straw mulch compared to the sourced cultivars exposed to natural snow conditions (Figure 5B). However, ca. 1.5-fold higher SER titers were noted in the dehiscing catkins of the sourced cultivars exposed to straw mulch compared to any combination of treatments and cultivars. 

Titers of MEL were significantly affected by all the fixed factors and their interactions except for cultivar (*p* _(1,100)_ = 0.6943) and its interaction with treatment (*p* _(1,100)_ = 0.0730). In the dormant buds of sourced cultivars exposed to natural snow conditions, ca. 1.5- and 3.5-fold higher MEL titers were noted compared to the locally adapted cultivars exposed to natural snow and straw mulch treatments, respectively. Exposure to straw mulch resulted in ca. 3-fold higher MEL titers in the sourced cultivars compared to the locally adapted cultivars (Figure 5C). Dormant catkins of the sourced cultivars exposed to natural snow conditions possessed ca. 6.5-fold lower MEL titers compared to any treatment and its cultivar combinations (Figure 5C). However, there were no differences noted in the dehiscing catkins.

### 2.5. Changes in the Expression of Indoleamine Pathway Genes—Snowmelt Percolation Study

To investigate the molecular changes involved in the indoleamine pathway during snowmelt percolation associated with flowering responses in locally adapted and sourced hazelnut cultivars, we used tissue from M, NS, P, and S treatments [17]. The four major genes (*TDC*, *SNAT*, *ASMT*, and *COMT*) involved in the biosynthesis of SER and MEL [21] were examined in this study at different growth stages of buds (dormant, vegetative, and flowering) and catkins (i.e., dormant and dehiscing) in both locally adapted and sourced cultivars.

**Figure 6:**
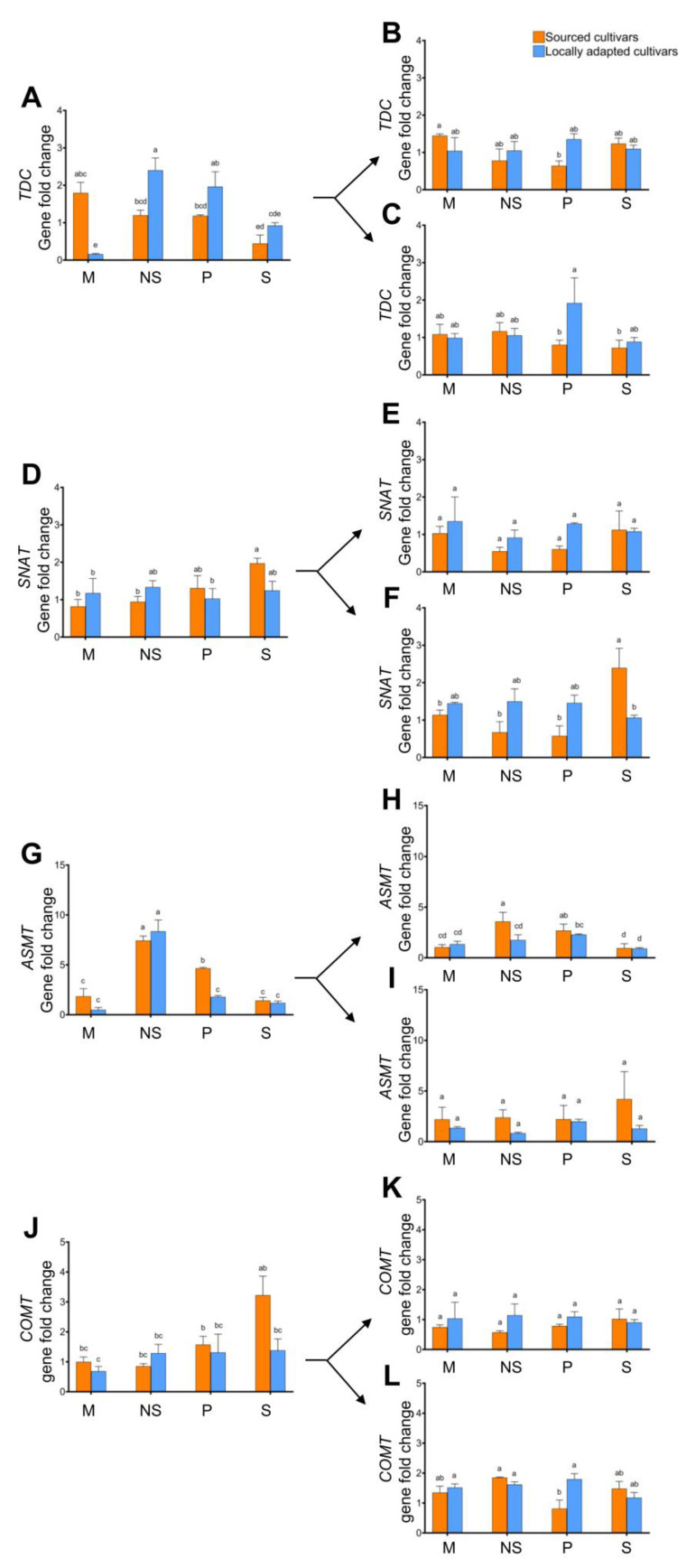
Effect of cold stress (abiotic) on expression levels of genes, viz. *TDC*, *SNAT*, *ASMT*, and *COMT*, in dormant ((**A**,**D**,**G**,**J**), respectively; initiated arrows), vegetative ((**B**,**E**,**H**,**K**), respectively; diverged up arrow), and flowering buds ((**C**,**F**,**I**,**L**), respectively; diverged down arrow) of sourced and locally adapted hazelnuts when exposed to P (plastic sheet that disallows snowmelt/water percolation), NS (no-snow; snow removed periodically until the following spring), M (porous plastic mulch sheet that allows water percolation), and S (naturally occurring snowfall). Different letters denote statistical significance in responses compared among the treatments (*p* < 0.05), and error bars represent SEM (fold-change with respect to locally adapted cultivar that received snow treatment) for that gene.

**Figure 7:**
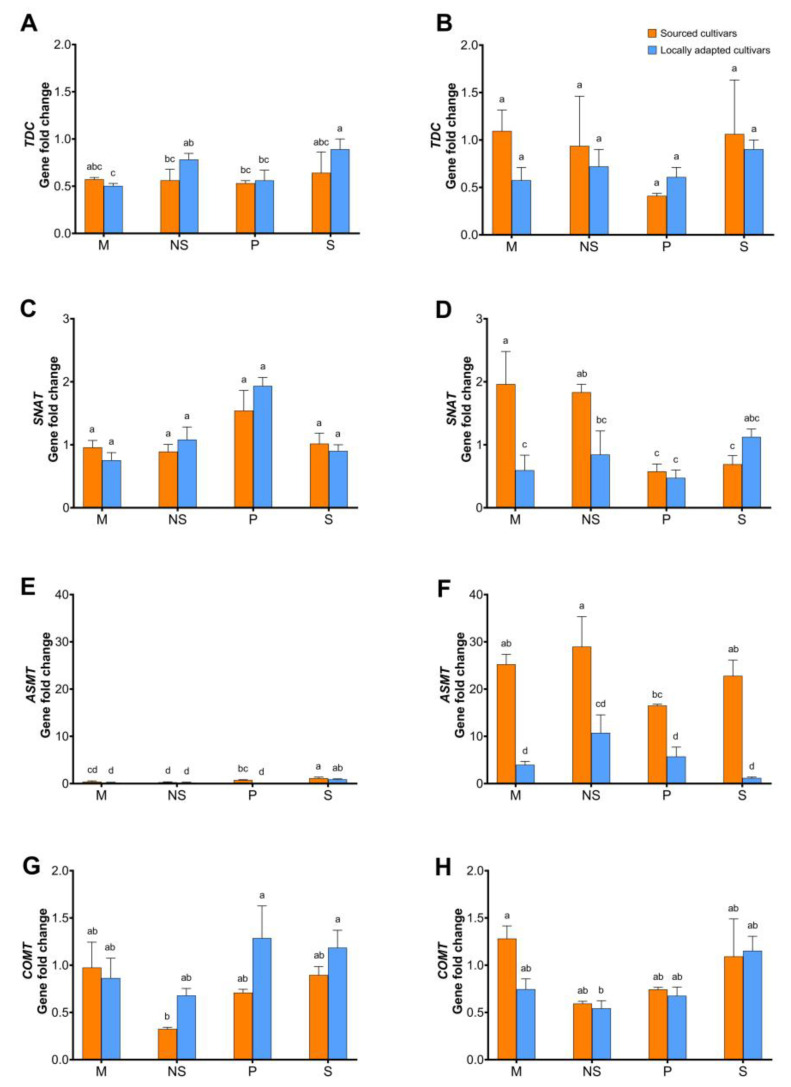
Effect of cold stress (abiotic) on expression levels of genes, viz. *TDC*, *SNAT*, *ASMT*, and *COMT*, in dormant ((**A**,**C**,**E**,**G**), respectively) and dehiscing ((**B**,**D**,**F**,**H**), respectively) catkins of sourced and locally adapted hazelnuts when exposed to P (plastic sheet that disallows snowmelt/water percolation), NS (no-snow; snow removed periodically until the following spring), M (porous plastic mulch sheet that allows water percolation), and S (naturally occurring snowfall). Different letters denote statistical significance in responses compared among the treatments (*p* < 0.05), and error bars represent SEMs (fold changes with respect to locally adapted cultivar that received snow treatment) for that gene.

In the dormant buds, there was an increasing trend (ca. 2-fold) in the expression of *TDC* in the locally adapted cultivars in response to NS and P treatments compared to locally adapted cultivars exposed to snow treatment (Figure 6A). The expression of *SNAT* was upregulated at least 3-fold in the sourced cultivars exposed to natural snow compared to its counterparts exposed to natural snow (Figure 6F). When dormant buds were assessed, *ASMT* expression levels were significantly upregulated in NS (ca. 7-fold), P (ca. 5-fold) treatments of sourced cultivars, and NS (ca. 9-fold) treatments of locally adapted cultivars compared to locally adapted cultivars exposed to natural snow (Figure 6G). When catkins were assessed for regulation of the above-listed genes, except *ASMT*, there were no changes in expression levels either among the different treatments of the sourced cultivars or within each treatment. The expression of *ASMT* in the dormant catkins significantly decreased in M, NS, and P of locally adapted cultivars and with M and NS treatments of sourced cultivars compared to its expression in locally adapted cultivars exposed to natural snow (Figure 7E). In the dehiscing catkins of sourced cultivars, a reverse trend of the *ASMT* expression in all treatments was noted, with an increase of ca. 20- to 30-fold changes compared to the locally adapted cultivars (Figure 7F). 

### 2.6. Changes in the Expression of Indoleamine Pathway Genes—Straw Mulch Study

The expression profiles of the above-mentioned genes involved in the indoleamine biosynthesis pathway were tested at different growth stages of buds (dormant, vegetative, and flower) and catkins (dormant and dehiscing) in both locally adapted and sourced cultivars. Tissue collected from two temporally different experiments, i.e., snowmelt percolation [17] and straw mulch (current study) blanketing, with the NS and P treatments, as opposed to the M and S treatments, and exposed to natural snow were used for further genetic analyses. In any given experiment, locally adapted cultivars exposed to natural snow were considered controls, and gene regulation data were normalized to them. 

**Figure 8:**
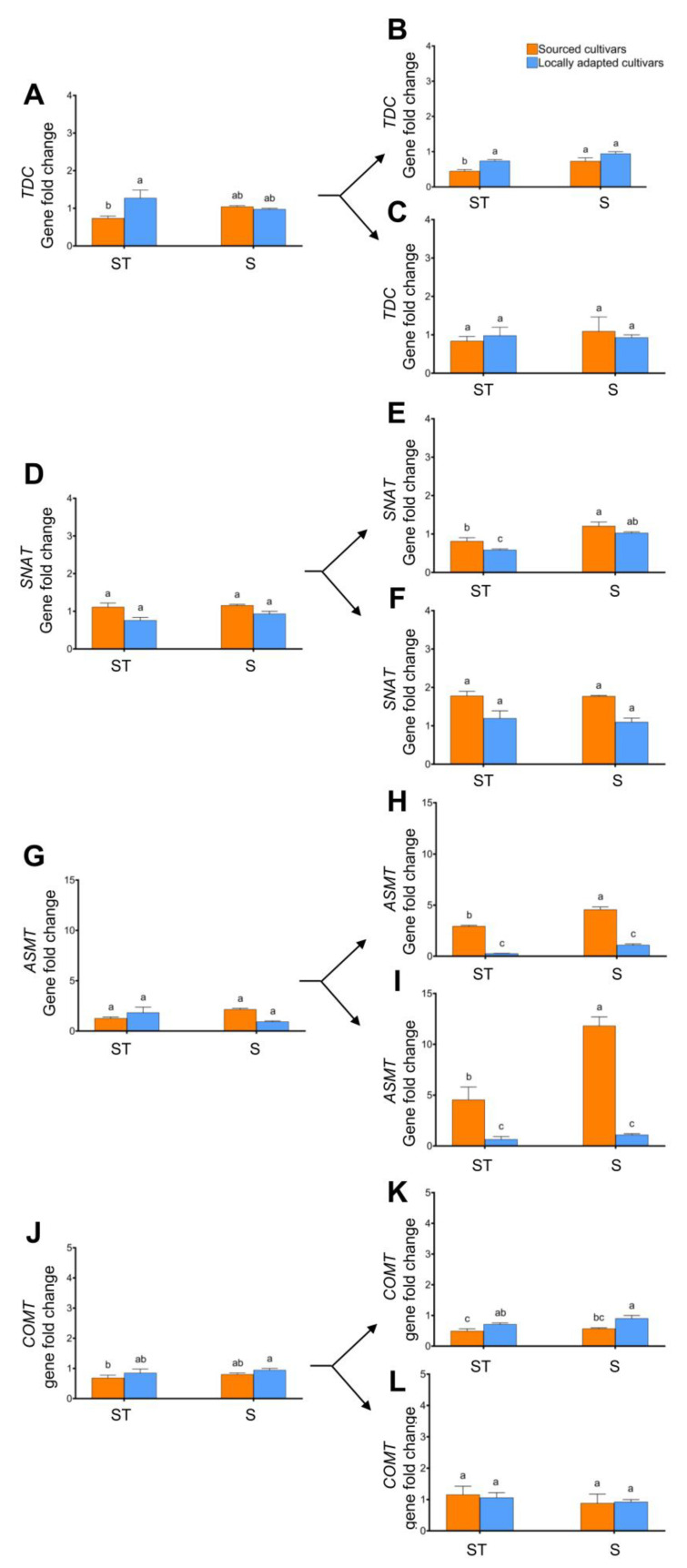
Effect of cold stress (abiotic) on expression levels of genes, viz. *TDC*, *SNAT*, *ASMT*, and *COMT*, in dormant ((**A**,**D**,**G**,**J**), respectively; initiated arrows), vegetative ((**B**,**E**,**H**,**K**), respectively; diverged up arrow), and flowering buds ((**C**,**F**,**I**,**L**), respectively; diverged down arrow) of sourced and locally adapted hazelnuts when exposed to wheat straw mulch and natural snow conditions. Different letters denote statistical significance in responses compared among the treatments (*p* < 0.05), and error bars represent SEMs (fold changes with respect to locally adapted cultivar that received snow treatment) for that gene.

The expression of *TDC* was similar with natural snow and straw mulch exposure. However, the *TDC* expression in the sourced cultivars blanketed with straw mulch was significantly lower compared to locally adapted cultivars with the same treatments (Figure 8A). There were no changes in gene regulation among the interactions when female flowers were analyzed for *TDC*, but 2-fold downregulation was noted when vegetative buds of the sourced cultivars exposed to straw treatment were analyzed. In the dormant or dehiscing catkins, no changes in the *TDC* regulation were noted among the treatments.

An opposite trend was noted in the expression of *SNAT* compared to *TDC*. Straw treatment revealed no significant effect on *SNAT* regulation in both sourced and locally adapted cultivars, except higher expression was noted in the vegetative buds of sourced cultivars irrespective of the treatment (Figure 8E). *ASMT* expression showed higher regulation in the sourced cultivar regardless of the treatment. There was at least 5- and 10-fold higher expression of *SNAT* in the female flowers of sourced cultivars exposed to straw mulch and natural snow conditions, respectively, compared to the locally adapted cultivars (Figure 8I), and in the vegetative buds, at least 2.5- and 5-fold higher expression was noted (Figure 8H). There were no notable changes in the expression of *COMT*, except ca. 2-fold downregulation was noted in vegetative buds of the sourced cultivars compared to the locally adapted cultivars exposed to natural snow conditions (Figure 8K). 

**Figure 9:**
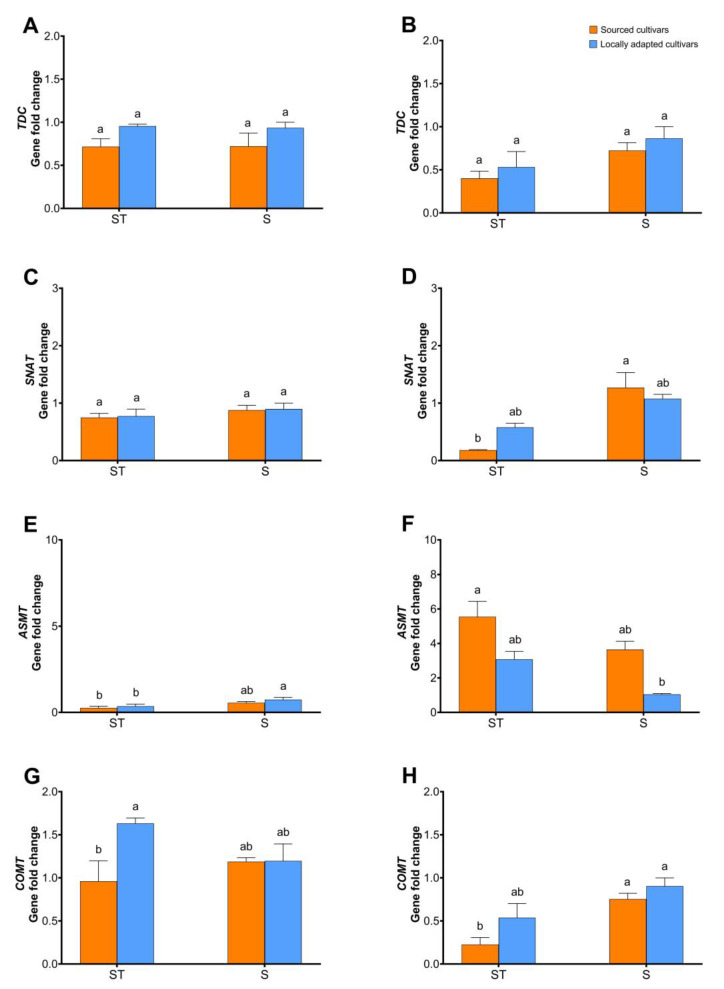
Effect of cold stress (abiotic) on regulation of genes, viz. *TDC*, *SNAT*, *ASMT* and *COMT*, in dormant ((**A**,**C**,**E**,**G**), respectively) and dehiscing ((**B**,**D**,**F**,**H**), respectively) catkins of sourced and locally adapted hazelnuts when exposed wheat straw mulch and natural snow conditions. Different letters denote statistical significance in responses compared among the treatments (*p <* 0.05), and error bars represent SEM (fold-change) for that gene.

Of all the genes studied here, the expression of *ASMT* in dormant and dehiscing catkins of sourced cultivars appears to be quite interesting (Figure 9E,F). When these trees were exposed to straw mulch, there was at least 2-fold downregulation and 5-fold upregulation in dormant and dehiscing catkins, respectively, in comparison to locally adapted trees exposed to natural snow conditions. In contrast, the dehiscing catkins of sourced cultivars exposed to straw mulch conditions showed downregulated expression of *COMT* by at least 5-fold compared to locally adapted cultivars exposed to natural snow conditions (Figure 9H).

## 3. Discussion

The two main objectives of the current study were: (1) to test the hypothesis that endogenous indoleamines, MEL and SER, in association with the regulation of indoleamine pathway genes would mediate the effect of horticultural solutions, such as application of wheat straw mulch, which assist in mitigating the erratic fluctuations during the reproductive phase of hazelnut cultivars; and (2) to create a ‘stress-busters’ toolkit comprising distinctive expression of indoleamine biosynthesis pathway genes, alongside the modulatory titers of TRP and its metabolites (SER and MEL) that confer abiotic stress resilience. The significant findings of this study are the following: (1) the use of straw mulch blanketing for cold-sensitive hazelnut trees can assist in abiotic stress (cold stress) alleviation during the reproductive phase; (2) SER accumulation assists in abiotic stress alleviation, and SER-associated *SNAT* gene responses align with the flowering patterns noted in the treatments; and (3) the *ASMT* gene appears to be a promising biomarker for understanding abiotic stress. 

### 3.1. Expression Patterns of Indoleamine Pathway Genes and Associated Metabolite Titers—Snowmelt Percolation Study

Our previous work demonstrated that the treatments in which snow was removed regularly to create a no-snow condition (NS) and the use of a solid plastic sheet (P) that did not allow for snowmelt/water percolation (Figure 1) induced harsh cold stress, along with reduced flowering and lower yield in the cold-sensitive sourced cultivars [17], which are not adapted to the continental cold climate of Ontario [17]. Amino acids have been shown to accumulate in overwintering temperate tress, especially TRP [22]. An increase in the expression level of *TDC* was found in the dormant buds exposed to the NS and P treatments, which metabolize TRP eventually to SER. These results align with the increase in SER content in the dormant bud that received NS and P treatments, particularly in the sourced cultivars [17]. SER accumulation could also deter/alter the *TDC* functionality and regulate the conversion of TRP to SER [13]. Further, locally adapted hazelnut trees undergoing more stress (accumulation of SER) with those treatments clearly demonstrated the effects of intervention in an otherwise tightly regulated natural phenomenon of endodormancy, as flowering was not affected [17]. This response conveys potential adaptability and the innate capacity to rapidly adjust to erratically fluctuating changes in the environment (here, abiotic stress due to NS and P treatments). Unchanged expression of *SNAT* could be due to the retention of SER, a ‘stress buster’ molecule, to counter abiotic stress. It is interesting to note the pattern of *SNAT* expression in the female flowers of the sourced cultivars mimicking flowering responses among the treatments, of which the S treatment was significantly higher than the other treatments, followed by the M treatment [17]. Here, SER titers noted in the female flowers possessed an inverse pattern to that of *SNAT*, clearly suggesting a role for SER as a flowering promoter (lower titers) in M and S treatments and as a ‘stress buster’ molecule in NS and P treatments (higher titers) [1,17]. This role probably emphasizes the resource allocation resulting in a poor female percentage favoring bare survival. *ASMT* and *COMT* regulate MEL biosynthesis from SER, probably via NAS (not in the scope of this study); hence, interplay during their regulation appears to be inverse to the percentage of female flowers responding in dormant buds (*ASMT*) and vice-versa (*COMT*). It is suggested that *ASMT* evolved into *COMT* during terrestrialization, and both are needed for MEL biosynthesis [23]. *ASMT* has been attributed to abiotic stress [24]. The absence of treatment effects on the titers of MEL suggested no clear inferences about flowering patterns [17]. However, *ASMT* expression appears to be an excellent biomarker for determining vegetative versus flowering bud differentiation. In this study, only one *ASMT* gene response was studied due to limitations in the annotation of the hazelnut genome. 

In the vegetative buds, an inverse pattern to that of the flowering responses probably suggests that the accumulation of SER, but not MEL, tends to program the bud to be female since all buds possess the potential gear to become a female flower (Figure 2). It is noteworthy that the locally adapted cultivars demonstrated a similar pattern in the dormant buds, but the expression of *ASMT* and *COMT* appeared to remain unchanged in vegetative or female flowers. The accumulation of MEL in female flowers of locally adapted cultivars exposed to P treatment suggests their adaptation to fluctuations and retaliation by resource allocation, with the flowering percentage not affected [17]. Tissue obtained from catkins possessed no clear patterns between genetic and biochemical endpoints except the expression of *SNAT* and *ASMT* in the dormant catkins of both the cultivars and *COMT* expression in the dehiscing catkins of both the cultivars. Surprisingly, the genetic expression in male reproductive organs possessing a similarity to the percentage female flowers despite being either protogynous (sourced cultivars) or protandrous (locally adapted), is quite intriguing. We questioned whether the treatment triggered similar effects in all parts of the tree. MEL titers in the dormant and dehiscing catkins of locally adapted cultivars clearly demonstrated a stress pattern per the treatments. This finding suggests that accumulation of MEL decreases flowering [25], probably at the cost of coping with stress. Interestingly, a similar pattern was noted when the catkin tissue was analyzed, and the pattern suggests that the regulation of *ASMT* is inverse to the female flower percentage and vice-versa for *COMT* expression.

MEL is a pleiotropic signal molecule that plays a critical role in regulating plant growth and development, as well as providing physiological protection against various environmental stresses [25,26,27]. MEL is known for its detoxification ability, by which it activates antioxidant enzymes, such as superoxide dismutase, catalase, etc., under various abiotic stress conditions [25]. Stress mitigation and resource allocation appeared to be modulated by the expression of genes involved in indoleamine biosynthesis, alongside their titers [17]. In the subsequent year, this study was advanced to identify simple and ecofriendly horticultural solutions and validate the ‘stress buster’ toolkit. Straw mulch provides the air-laden blanketing effect to minimize the erratic fluctuations affecting the root zone during the sensitive reproductive phase of hazelnuts [28,29]. We used the most stressed treatment trees from both the cultivars to apply straw mulch, anticipating stress revival and improved performance. 

### 3.2. Percent Female Flowers, Expression Patterns of Indoleamine Pathway Genes and Associated Metabolite Titers—Straw Mulch Study

The percentage of female flowers was on par among the cultivars and treatments, with a slight increase in the sourced cultivars exposed to straw mulch (ST), and this treatment could be a simple horticultural practice for the trees in the ground [30] for coping with abiotic stress due to relocation to a new agroclimatic zone. The trees used for the ST treatment were exactly those from the NS and P (harsh treatment) treatments of the preceding year’s experiments; hence, our objective of obtaining similar flowering responses as those of the natural snow (SN) treatment (preceding years’ M and S treatments) [17] was met. There could be other potential factors that were not addressed or that were not within the scope of this study, such as microbial interactions, ion retention, etc., with the use of ST [28,29], which could have led to revival, in addition to the anticipated thermal blanketing and gradual snowmelt percolation through it [30]. Additionally, the impact of natural snow (SN) on either cultivar appeared to be similar to that in the previous study (S treatment) [17]. ST had no effect on the percentage of female flowers of locally adapted cultivars, whereas the sourced cultivars benefitted from the extra layer of less-dense snow simulated with straw mulch. Interestingly, most of the genetic responses in this study were minimal and/or unchanged except for *ASMT*. As noted in the previous study, *ASMT* responses appear to dictate the fate of the bud. It appeared to be pronounced in the cold susceptible sourced cultivar flowers, in which a 2-fold decline in the expression of *ASMT* determined an increased chance of them yielding flowers. This finding is evident from SER titers in the flowers of sourced cultivars, in which the SN group possessed higher titers compared to the ST group, reflecting a slight increase in flowering percentage. 

In the vegetative buds, the expression of *TDC* was lowest in the sourced cultivars exposed to ST, suggesting accumulation of TRP [31]. No significant changes in gene expressions were noted when catkins were assessed. Cold stress significantly increased the concentration of MEL in the reproductive hazelnut buds [17]. SER and MEL were found at the highest levels in the least developed flower buds with decreasing concentrations as the flower buds matured, as noted in datura [1]; moreover, the increase in MEL levels in the flowering buds was also found to decrease flowering [25]. Together, these data indicate that the reduction in indoleamine biosynthesis-related genes in the reproductive tissues of the sourced cultivars might be involved in the increase in the flowering rate in response to ST treatment. Decreases in MEL have been observed with increased flowering in other species, such as apple [32] and datura [1]. 

## 4. Materials and Methods

### 4.1. Reproductive Phase Development in Hazelnut

Hazelnuts are monoecious, wind pollinated, and self-incompatible in nature [33]. Hazelnuts are dichogamous, as the male (catkins, staminate) and female (pistillate) flowers are separate (Figure 2), and they mature at different times. Temporal samples of male and female reproductive organs were collected from a mature orchard and dissected under a microscope. Images of temporally collected, dissected floral, and vegetative organs are displayed in Figure 2. 

### 4.2. Field Experiment—Snowmelt Percolation Study (Figure 1)

Experiments occurred in a hazelnut cultivar trial plot in a gently sloped field (1–4%) at the University of Guelph, Simcoe Research Station, in Simcoe, ON, Canada (latitude 42°83′N, longitude 80°30′W, elevation 200 m). The soil type is Fox sand, with a low organic matter content (1.2%) [16]. The detailed snowmelt percolation experiment has been discussed elsewhere (Figure 1) [17]. Briefly, the genotypes comprised different cultivars and selections from Europe and Oregon (called sourced cultivars) and New York and Ontario (called locally adapted cultivars), depending on the source of origin/breeding [34]. Cultivars were grouped so that there were mulch (M), no-snow (NS), plastic (P), and snow (S) treatment groups with three trees per treatment, resulting in a total of 24 trees with 12 trees/block (Figure 1). A total of eight twigs were selected for the sample collection, having two twigs in each direction; further, each twig consisted of 10 buds and at least two to three catkins. Snow was constantly removed manually with shovels right from the initial occurrence of the first snow fall until the end of snowfall for the no-snow treatment (NS). Plastic sheets were used to disallow the snowmelt percolation in the plastic (P) treatment, and sheets with pores, called plastic mulch (M) treatment, allowed for percolation. The control treatment consisted of natural snow (S) without any disturbance for the entire period of the experiment, which allowed for snowmelt percolation. 

### 4.3. Field Experiment—Straw Mulch Study (Figure 1)

The results from the prior experiment suggested that an extra blanket of straw mulch underneath the snowfall should assist the sourced cultivars in mitigating erratic fluctuations during critical flowering stages. This finding led to our designing a follow-up experiment, aiming to understand thermoregulation, in addition to water percolation. Here, tress labeled NS and P were exposed to wheat straw mulch (ST) (ca. 30 cm), and those labeled M and S were exposed to natural snow (SN) conditions (Figure 1). Otherwise, the experimental design, endpoints assessed, and sample collection were similar to the above-described experiment [17]. Briefly, eight twigs (two per direction) from each tree possessing 10 buds and two to three catkins were tagged and labeled. There was a total of 24 trees with 12 per block (sourced versus locally adapted) and per treatment included in this study.

### 4.4. Sample Collection

Sample tissues from mulch and straw experiments were broadly classified as catkins and buds and were collected over two time periods [17]. The first time point was when catkins were in the endodormancy stage, and the second time point was when the catkins were shedding the pollen; hence, they were called dehiscing catkins (>50% shedding). Dormant bud samples were collected first at the endodormancy stage and then as flowering buds (buds in which almost >50% of the style protruded). Vegetative buds were collected at the same time and were recognized as buds that did not possess pink tips or styles. Samples were collected using a destructive sampling methodology for both time points. Using pruners, the buds and associated axil wood ca. 5 mm from the base of the bud were chopped and collected into sterile 15-mL centrifuge tubes. The catkins were chopped similarly. During the second time period, using destructive sampling, female flowers and vegetative buds were collected separately into 15-mL sterile centrifuge tubes. For pollen dehiscing catkins, the entire catkin was collected, avoiding any adjacent buds. The samples were stored at −80 °C until processed. 

### 4.5. Separation of Metabolites and Metabolite Detection (UPLC-MS)

Samples were ground into fine powder in a mortar and pestle using liquid nitrogen. The samples (ca. 100 mg) were then suspended in 0.5 mL of extraction solvent, which comprised 75% methanol (MS Grade, Fisher Scientific, Ottawa, ON, Canada; MeOH) and 4% formic acid (MS Grade, Fisher Scientific, Canada) in Milli-Q water. The samples were then held at −20 °C for 30 min and spun down (15 min, 4 °C, 14,000 rpm), and the supernatant was removed. A second extraction was performed on the same sample using conditions similar to those described above, and the supernatants were pooled. Solid phase extraction (Oasis**^®^** HLB 1cc (30 mg), Waters Canada, Mississauga, ON, Canada) was deployed to concentrate the samples before eluting them in 200 µL of methanol. Later, the elutant was then filtered through a 0.22-μM centrifuge filter (Millipore, Sigma, Oakville, ON, Canada; 1 min, 13,000 rpm). All standards were analytical grade and purchased from Sigma Aldrich, Canada. Phytohormones were separated by reverse phase liquid chromatography (ultra-performance liquid chromatography system (UPLC); LC-40D XS, Shimadzu, Japan) by injection of a 5-μL aliquot of sample onto a Shim-pack Scepter LC column (2.1 × 50 mm, 1.9 μm; Mandel Scientific Company, Guelph, ON, Canada). Metabolites were separated with a gradient of solvents A (0.1% formic acid) and B (100% methanol) with initial conditions at 95% A (5% B) increased to 5% A (95% B) over 4 min using a curve of 0. The column temperature was 40 °C, and the flow rate was 0.2 mL/min. Metabolite peaks were identified by comparison to standards and quantified by a standard curve generated using a similar separation method and gradient conditions. Phytohormones were detected using a single quadrupole mass spectrometer (LCMS 2020, Shimadzu, Japan) in single ion recording mode (SIR). TRP, SER, and MEL were detected in positive mode with cone voltages of 10, 10, and 15, respectively, for mass to charge (*m*/*z*) of 205, 177, and 233, respectively. In all cases, the probe temperature was set to 250 °C with a gain of 5; the capillary voltage (positive and negative) was set to 0.5 kV. The instrument limits of detection were 6.1 pg/mL, 24.4 pg/mL, and 9.3 pg/mL for TRP, SER, and MEL, respectively, and the method detection limits were found to be 0.41 ng/g, 1.62 ng/g, and 6.17 ng/g, respectively. The linear ranges for TRP, SER, and MEL were 24.4 ng/mL–25 μg/mL, 97.7 ng/mL–25 μg/mL, and 38.1 pg/mL–6.25 μg/mL, respectively.

### 4.6. Identification of Orthologous Gene Sequences

The indolamine-related gene sequences were retrieved using *Arabidopsis thaliana* as a reference genome. Because of high sequence divergence between *Arabidopsis* and *C. avallena*, it was not feasible to obtain sequences directly. Therefore, two bridging species (*Populus tricocarpa* and *Vitis vinifera*) were used to find the sequences in *C. avallena*, where *Arabidopsis* gene sequences (*AtTDC* (AJ011049.1), *AtSNAT* (At1g32070), *AtASMT* (AT4G35160), *AtCOMT* (AY081565)) were used to retrieve orthologous gene sequences from sequenced and annotated genomes of *Populus spp*. (*Populus tricocarpa* (*PtTDC* (MK440567.1), *PtSNAT* (XM_006388031), *PtASMT* (XM_002312897), *PtCOMT* (XM_002317802)) and *Vitis vinifera* (*VvTDC* (XM_002280249.4), *VvSNAT* (XM_002266325), *VvASMT* (KC517477.1), *VvCOMT* (XM_003634113)) using the tblastn and blastn tools from the National Center for Biotechnology Information (NCBI) with default settings. The resultant sequences were used as queries to identify the orthologous sequences in the *C. avellana* Jefferson genome and in transcriptome databases (https://www.cavellanagenomeportal.com/, accessed on 24 April 2020) [35,36]. The sequences with top queries were used to design the primers, and *actin* was identified from *C. heterophylla* [37]. The designed primer pairs for the tested genes are listed in Table 1. 

### 4.7. RNA Extraction, cDNA Synthesis and Gene Expression Analysis 

To examine the differential expression of the selected genes, total RNA from catkin and bud samples was extracted using a CTAB protocol [38]. All RNA extracts were treated with DNase using the RNase-free DNase set (Qiagen, Mississauga, ON, Canada) and then purified using a RNeasy Mini Kit (Qiagen, Mississauga, ON, Canada). The cDNA was synthesized from about 2 μg of DNase-treated RNA using the SuperScript^®^ VILO™ cDNA Synthesis Kit (Invitrogen, Burlington, ON, Canada) in a total volume of 20 μL. The cDNA was diluted 1:10 with ultrapure water.

Quantitative real time-PCR (qRT-PCR) was performed using the CFX connect real-time Detection System (Bio-Rad, Mississauga, ON, Canada) and SsoFast™ EvaGreen^®^ Supermix (Bio-Rad, Mississauga, ON, Canada) using gene-specific primers (Table 1) for three biological and three technical replicates for each sample. The reaction was established using the manufacturer’s instructions. The expression of each gene was normalized to that of actin and was calculated relative to the locally adapted cultivar that received snow treatment (control). Normalized relative fold expression was calculated using CFX manager software, version 3.1 (Bio-Rad, Mississauga, ON, Canada) according to the 2^−∆∆CT^ method [39]. 

### 4.8. Data Analyses

For the phenological endpoints, the experiment was a randomized complete block design, with the treatment being the main factor of interest. For each treatment, temporal readings of phenological events were noted in the locally adapted and sourced hazelnut cultivars, which was considered the blocking factor. For all responses, the normal distribution and constant variance assumptions on the error terms were verified by examining the residuals. For flowering data, treatment, and/or block effects (*p*-value < 0.05), the least square (LS) means were separated at the α = 0.05 level. To facilitate understanding, means ± SEMs are presented graphically. The GLIMMIX procedure in SAS software was used to complete the analyses. In the phytohormone analyses, there were three replicates per treatment, and each treatment comprised two blocks. Treatments were fixed factors, and the metabolite responses (ng/g FW) were subjected to ANOVA using PROC GLIMMIX in SAS^®^ Studio software (SAS Institute Inc., Cary, NC, USA). For all responses, the normal distribution and constant variance assumptions on the error terms were verified by examining the residuals. When the effects were significant, LS means were separated at α = 0.05 level. Means ± SEM (ng/g FW) responses per each metabolite for three sites are presented in graphical format.

The GLIMMIX procedure in SAS^®^ Studio software (SAS Institute Inc., Cary, NC, USA) was used to complete the genetic analyses. In the analyses, there were two cultivars with two (straw mulch study) or four (snowmelt percolation study) treatments per cultivar tested as fixed effects, and three replicates per treatment were tested as a random effect. Means were subjected to analysis of variance (ANOVA) when the effects were significant, and LS means were separated at α = 0.05 level. Testing of normality was performed before any analysis. Means ± SEM expression responses for each gene are presented in a graphical format. 

## 5. Conclusions

Temperature fluctuations during the late winter in many regions, such as southern Ontario, negatively affect flowering and eventually the performance of desired sourced hazelnut cultivars of interest to industry. These most desired cultivars produce at least 10-fold lower yields than in the location where they were bred [16]. Hazelnut research comparing the performance of locally adapted and sourced cultivars for their female flower ratio and yield has been limited [16,17,40]. The climate crisis has been progressing at a faster pace than before and ought to be curtailed, as events, such as freezing rain in the place of snow, denser snow, or no snow, could be detrimental to plant development processes, especially during flowering periods. Reviving from extreme stress (freezing winters), trees tend to invest in reproduction for perpetuation of their species, and the erratic fluctuations mentioned above could hamper vital processes and trigger poor performances, as the trees tend to enter bare survival mode due to resource allocation. 

Our results showed that cold (abiotic) stress probably has triggered changes in the expression of genes involved in indoleamine pathway (*TDC*, *SNAT*, *ASMT*, and *COMT*) including titers of TRP and its metabolites (SER and MEL). Locally adapted cultivars coped with absence of snow (NS) and absence of water percolation (P) treatments by regulating those genes and titers of metabolites involved in the indoleamine pathway. Sourced cultivars revived from the harsh treatments (NS and P), when exposed to straw mulch treatment (ST), produced female flowers at a higher rate than the locally adapted cultivars exposed to snow treatment. This extra blanketing probably provided thermal protection and allowed for water percolation, which assisted in improved flowering responses in the sourced cultivars. Similar responses noted in the male reproductive organs (catkins) suggest that the entire tree was probably programmed to manage such erratic fluctuations. 

This omics-driven approach has identified simple and ecofriendly horticultural solutions that should mitigate the erratic fluctuations during the reproductive phase of hazelnut cultivars. A toolkit comprising the distinctive expression of indoleamine biosynthesis pathway genes, alongside the modulatory titers of TRP and its metabolites (indoleamines), could assist in understanding stress resilience in newly introduced cold-sensitive cultivars of hazelnut. Extrapolation of this technology to other temperate trees could assist in alleviating reproductive depression. 

## Figures and Tables

**Table 1:** List of primers used in qRTPCR.

Gene Name	Forward	Reverse
***TDC***	GCCCGGTTATCTTCGTGGAA	TGGGCTTTGCCAATGGGTTA
***SNAT***	GCGATCATATGGGACGTGGT	TCCCTTTGGATGACAGCTCG
***ASMT***	GCAAACGTTGGTGAAGGCAT	ACCCCCGACATGTTCAACTC
***COMT***	CACCGGCACTTTCCTCTCAT	GCTTAGCATCCGGTCCAGAA
***Actin***	GATGATGCTCCAAGGGCAGT	TTTCGACTGGGCCTCATCAC

## Data Availability

Not applicable.
